# Factors Associated With HIV Infection in Zimbabwe Over a Decade From 2005 to 2015: An Interval-Censoring Survival Analysis Approach

**DOI:** 10.3389/fpubh.2019.00262

**Published:** 2019-09-18

**Authors:** Rutendo Birri Makota, Eustasius Musenge

**Affiliations:** Division of Epidemiology and Biostatistics, Faculty of Health Sciences, School of Public Health, University of the Witwatersrand, Johannesburg, South Africa

**Keywords:** interval-censoring, HIV, survival, prevalence, Zimbabwe

## Abstract

**Objectives:** The main objective of this study was to compare results from two approaches for estimating the effect of different factors on the risk of HIV infection and determine the best fitting model.

**Study design:** We performed secondary data analysis on cross-sectional data which was collected from the Zimbabwe Demographic Health Survey (ZDHS) from 2005 to 2015.

**Methods:** Survey and cluster adjusted logistic regression was used to determine variables for use in survival analysis with HIV status as the outcome variable. Covariates found significant in the logistic regression were used in survival analysis to determine the factors associated with HIV infection over the 10 years. The data for the survival analysis were modeled assuming age at survey imputation (Model 1) and interval-censoring (Model 2).

**Results:** Model goodness of fit test based on the Cox-Snell residuals against the cumulative hazard indicated that Model 1 was the best model. On the contrary, the Akaike Information Criterion (AIC) indicated that Model 2 was the best model. Factors associated with a high risk of HIV infection were being female, number of sexual partners, and having had an STI in the past year prior to the survey.

**Conclusion:** The difference between the results from the Cox-Snell residuals graphical method and the model estimates and AIC value maybe due to the lack of adequate methods to test the goodness-of -fit of interval-censored data. We concluded that Model 2 with interval-censoring gave better estimates due to its consistency with the published results from literature. Even though we consider the interval-censoring model as the superior model with regards to our specific data, the method had its own set of limitations.

## Introduction

The 90–90–90 targets was launched by the Joint United Nations Programme on HIV/AIDS (UNAIDS) and partners with the aim to diagnose 90% of all HIV positive persons, provide antiretroviral therapy (ART) for 90% of those diagnosed, and achieve viral suppression for 90% of those treated by 2020 (1).

In Zimbabwe, a population based survey carried out in 2016 reported that 74.2% of people living with HIV (PLHIV) aged 15–64 years knew their HIV status. Amoung the PLHIV who knew their status, 86.8% self-reported current use of Antiretroviral treatment (ART), with 86.5% of those who self-reported, are virally suppressed (2). In order for these 90–90–90 targets to be met, prevalence, and incidence rates estimates are crucial in understanding the current status of the HIV epidemic and determine whether the trends are improving in a manner which can facilitate to achieve the 2020 target.

The gold standard for estimating the HIV incidence is to test uninfected individuals for new infections periodically, however this method is feasible though costly and time-consuming. In addition, even if an HIV negative cohort is followed over-time, the exact date of infection is rarely observed (3). In this scenario, an interval can be determined between the latest negative and the earliest positive test dates. Taking into consideration the issue of cost and time, cohort analysis for estimating the HIV incidence rate in a general epidemic will not produce estimates which are representative of the whole population. Due to these reasons, sentinel surveillance systems have been set up to monitor the spread of the pandemic (4). In addition, population-based surveys, in which HIV tests are performed, are carried after every 5-years in Zimbabwe. The advantage of the population-based survey is that, data is more representative of the population than a cohort. On the other hand, the same data does not provide the exact date of the infection but rather provide what is called “current status data.”

Current status data occurs when an individual is observed at one single point, and the only information obtained is whether the event of interest has occurred (5). An example of current status data includes information collected during a demographic health survey, in which an individual is tested whether they are HIV positive or negative. If an individual were found to be HIV positive, the individual was recorded as left censored at the time the test was done. If an individual were HIV negative, they were recorded to be right censored. Sometimes current status data can be referred to as case interval-censored data, with case II interval-censored data referred to as the general case (6). Interval censoring takes into account the range, that is, an interval inside of which one can say the outcome of interest has occurred (7). Given that we would want to determine the factors associated with the hazard of infection using data from these surveys, then survival analysis can be implemented.

In the setting of standard survival analysis, modeling the hazard rate of HIV infection can be achieved by imputing the time-to-onset of disease as the time at diagnostic visit (3). Modeling current status data using the mentioned two approaches may overestimate the hazard rate. However, analyzing this type of data using interval censoring will be a better approach. Although they are documented advantages of interval censoring compared to the standard cause-specific survival model, the superiority of the interval-censoring remains unclear in estimating the effect of different exposures on the risk of HIV infection. With this argument in mind, the main objective of this study was to compare results from these different approaches for estimating the effect of different factors on the risk of HIV infection and determine the best fitting model.

## Materials and Methods

### Study Design and Area

This study used data from Zimbabwe, a landlocked country, bordered by Mozambique on the East, South Africa on the South, Botswana on the West, and Zambia on the North and Northwest. Zimbabwe is sub-divided into 10 Provinces which are: Matabeleland South, Matabeleland North, Mashonaland East, Mashonaland Central, Mashonaland West, Midlands, Masvingo, Manicaland, Harare, and Bulawayo. Each province is subdivided into districts, and each district is made up of wards. The designs for the three 5 yearly surveys were cross-sectional.

### Zimbabwe Demographic Health Survey Data and Sampling

Demographic and Health Surveys (DHS) are nationally-representative household surveys that have been implemented in approximately 70 countries since 1984 (8–10). They provide data for a wide range of monitoring and impact evaluation in the areas of population, health and nutrition. Data used for the analysis were obtained from 2005–06, 2010–11, and 2015 ZDHS and were retrieved from the DHS programme website (https://dhsprogram.com) (11). A representative probability sample of 10,800, 10,828, and 11,196 households were selected for the 2005–06, 2010–11 and 2015 ZDHS, respectively. A two-stage cluster sampling technique was used to select the households. The first stage selected 400, 406, and 400 enumeration areas (EAs) for 2005–06, 2010–11, and 2015 ZDHS, respectively. At the second stage, using a complete listing of households in the selected EAs, a fixed number of households were randomly chosen. This allowed the use of EAs specific weights to be assigned in the design (8–10).

### Measurement of the Outcome (HIV Status) and Explanatory Variables

With consent from the respondent or parent/guardian (for minors), blood samples were collected in all households for HIV testing in the laboratory for females aged 0–49 and males aged 0–54. Blood spots were collected on filter paper from a finger prick and transported to a laboratory for testing. An initial ELISA test was performed, and then retesting of all positive and 5–10 % of the negative tests with a second ELISA was done. If they were discordant results on the two ELISA tests, a new ELISA or a Western Blot was performed. The data used for this study was obtained from the DHS Data Archives (11) and only included individuals aged 15–49 years. The following explanatory variables were extracted from the dataset: sex, marital status, education level, religion, currently employed, place of residence, STI treatment in the past 12 months and number of sexual partners (8–10).

### Ethical Considerations

The ZDHS HIV testing protocol for all the three surveys was reviewed and approved by the ethical review boards Medical Research Council of Zimbabwe (MRCZ) in Harare, Zimbabwe; the ORC Macro Institutional Review Board in Calverton, Maryland, USA; and the Centers for Diseases Control (CDC) in Atlanta, Georgia, USA (8–10). This work was granted ethical clearance by the University of Witwatersrand's Human Research Ethics Committee (Medical) (No. M151154). The dataset used in this study was obtained through an application made to Measure DHS program, which was approved on the 16th of May 2017. The DHS Program is authorized to distribute, at no cost, unrestricted survey data files for legitimate academic research. Registration was required for access to data.

### Statistical Methodology

#### Application to Zimbabwe Demographic Health Survey

**Figure 1:**
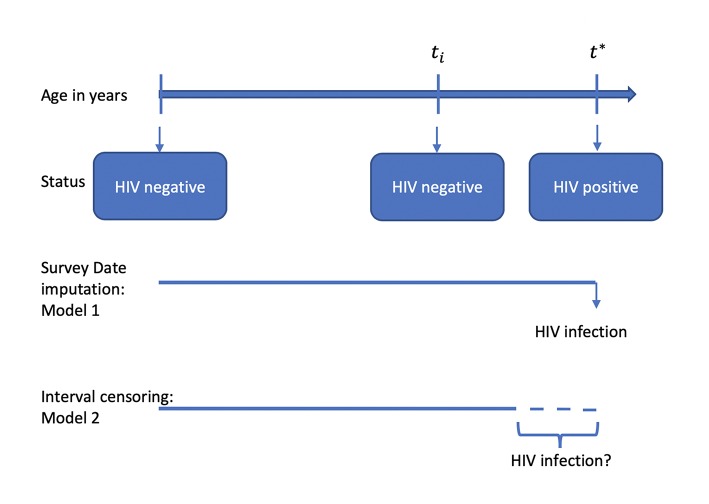
Illustration of different modeling strategies when investigating the factors associated with HIV infection.

The socio-demographic datasets for men and women records were appended to provide a single analysis dataset for all the three surveys. The appended dataset was then merged using the unique combination of the individual line number, household line number, and cluster (EA) number to the HIV prevalence dataset. All individuals without an HIV test result, never been sexually active, and individuals who did not have an age at first intercourse were excluded from the analysis. In the case of individuals who were HIV positive when the survey was conducted, the age at HIV infection was defined as age at survey date and for individuals who were HIV negative, the age at HIV infection was right-censored at the date of survey in Models 1 (Figure 1). Accounting for interval-censoring, the age at HIV infection was interval-censored between age at first sexual intercourse and age at date of survey, but right-censored at the age at survey, for individuals who were HIV negative for Model 2. All the models assumed a parametric Weibull distribution for the baseline hazard λ_0_(*t*) which allowed estimation of β which is the vector of regression coefficient. Model Specification for all the models, refer to Supplementary Data.

### Statistical Analysis

In this study, the outcome or response variable was the HIV status, a binary variable. The study investigated the socio-cultural, socio-economic, behavioral, and demographic factors, which are associated with HIV. Trends in HIV prevalence were assessed using the non-parametric trend test in STATA. A stepwise logistic regression approach was adopted in STATA SE version 15.1 statistical software (12) using the command **svy: swaic** (13) on some selected explanatory variables highlighted earlier. Factors that were significantly associated with HIV from the stepwise survey logistic regression were then considered for the parametric survival analysis. The most suitable baseline hazard function was investigated using the package **icenReg** (14) in R software. The data were modeled assuming age at survey imputation and interval-censoring (Figure 1). The model goodness-of-fit (GOF) test was assessed using the Akaike Information Criterion (AIC) and two graphical methods which included the Cox-Snell residuals. All analysis were performed in STATA SE version 15.1 and R statistical software.

## Results

**Figure 2:**
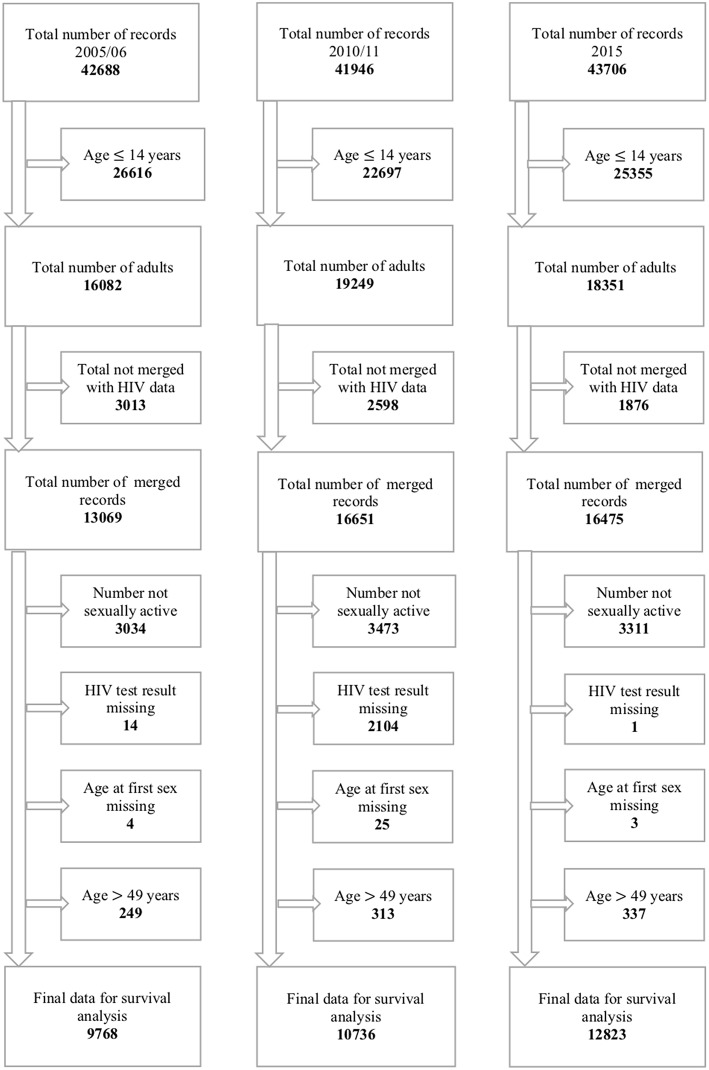
Flowchart of records in the 2005–06, 2010–11, and 2015 ZDHS.

The 2005–06 ZDHS database had 10,800 households in which 42,698 records were retrieved. Of the 42,698 records, 16,082 records were for individuals aged 15–49 years with 7,175 (44.6%) males and 8,097 (55.4%) females. The 2010–11 ZDHS database had 10,828 households in which 41,946 records were retrieved. Of the 41,946 records, 16,651 records were for individuals aged 15–49 years with 7,480 (44.9%) males, and 9,171 (55.1%) females. The 2015 ZDHS database had 11,196 households in which 43,706 records were retrieved. Of the 43,706 records, 18,351 records were for individuals aged 15–49 years with 8,396 (45.8%) males and 9,955 (54.2%) females. The above information is represented in Figure 2.

Nationally, the non-parametric trend test (*p* < 0.001) showed a significant decline of HIV prevalence from 22.4 to 19.6 to 17.7% for 2005–06, 2010–11, and 2015 ZDHS, respectively (see Table 1). A similar decline trend was observed for gender, marital status, place of residence, education level, current employment status, STI in the past year preceding the survey and number of sexual partners.

**Table 1:** HIV prevalence in Zimbabwe and changes in HIV prevalence (weighted) from the Zimbabwe DHS surveys 2005–06, 2010–11, and 2015.

**Survey year**	**2005/6**	**2010/11**	**2015**	***p*-value**
**Characteristic**	**Prevalence (CI)**	**Prevalence (CI)**	**Prevalence (CI)**	
National summary	22.4 (21.6, 23.2)	19.6 (18.8, 20.3)	17.7 (17.0, 18.4)	<0.001
**Sex**				
Male	18.2 (16.7, 19.4)	16.2 (15.1, 17.3)	13.6 (12.7, 14.5)	<0.001
Female	25.1 (24.0, 26.2)	21.8 (20.8, 22.8)	20.7 (19.8, 21.6)	<0.001
**Marital status**				
Never married	10.8 (9.36, 12.3)	9.66 (8.29, 11.0)	7.86 (6.78, 8.95)	0.001
Married/cohabiting	20.5 (19.5, 21.5)	17.8 (16.9, 18.7)	16.8 (16.0, 17.6)	<0.001
Separated/divorced/widowed	45.6 (43.0, 48.2)	42.2 (39.6, 44.8)	38.3 (35.9, 40.8)	<0.001
**Place of residence**				
Urban	23.7 (22.2, 25.3)	22.0 (20.5, 23.4)	18.3 (17.3, 19.3)	<0.001
Rural	21.8 (20.8, 22.8)	18.5 (17.7, 19.4)	17.3 (16.4, 18.1)	<0.001
**Education level**				
No education/primary	22.6 (21.3, 23.9)	21.1 (19.7, 22.4)	20.7 (19.3, 22.1)	0.05
Secondary	22.6 (21.6, 23.7)	19.4 (18.5, 20.3)	17.4 (16.5, 18.2)	<0.001
Higher	16.3 (12.6, 20.0)	12.6 (9.86, 15.4)	12.1 (10.3, 13.9)	0.05
**STI treatment in past 12 months**				
No	21.4 (20.7, 22.2)	18.9 (18.2, 19.7)	17.2 (16.5, 17.8)	<0.001
Yes	46.2 (41.2, 51.2)	39.0 (34.0, 44.0)	37.2 (32.0, 42.3)	0.01
**No. of sexual partners**				
0	31.3 (28.9, 33.6)	28.2 (25.9, 30.5)	24.5 (22.2, 26.9)	<0.001
1	20.8 (19.9, 21.7)	18.2 (17.4, 19.1)	17.0 (16.2, 17.7)	<0.001
2+	19.5 (16.1, 22.9)	17.7 (14.8, 20.7)	15.3 (13.2, 17.4)	0.03
**Currently employed**				
No	22.7 (21.5, 23.9)	18.9 (17.9, 20.0)	17.9 (16.9, 19.0)	<0.001
Yes	22.1 (21.0, 23.2)	20.2 (19.2, 21.3)	17.5 (16.7, 18.4)	<0.001

*p-values below 0.05 are considered statistically significant.

The mean survival time for age at HIV infection for Model 1 was 41.1 years for females, 42.8 years for males in 2005/06 ZDHS; 41.9 years for females, 43.4 years for males in 2010/11 ZDHS and 42 years for females, 44 years for males in 2015 ZDHS. The mean survival time for age at HIV infection for Model 2 was 24.9 years for females, 28.3 years for males in 2005/06 ZDHS; 25.7 years for females, 29.4 years for males in 2010/11 ZDHS and 26.5 years for females, 30.5 years for males in 2015 ZDHS. According to the survival times, Model 2 produced lower times of survival before HIV infection.

**Figure 3:**
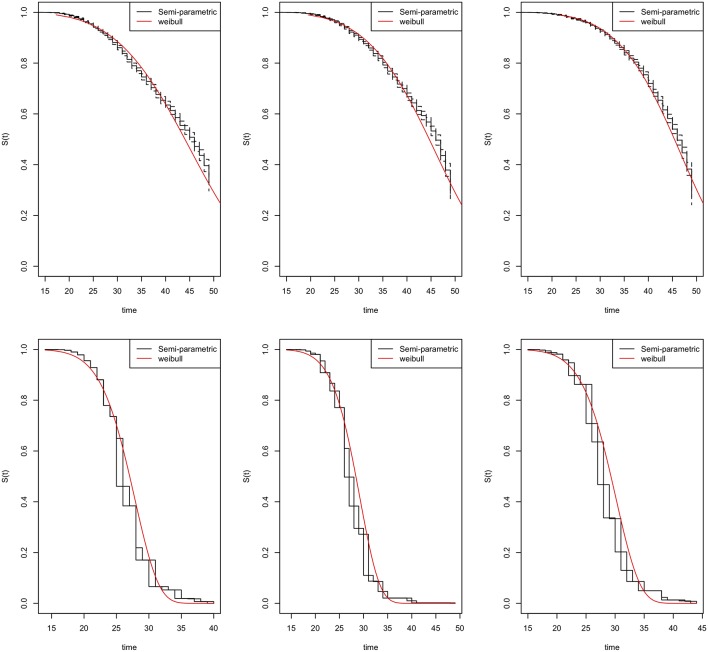
Goodness-of-fit tests for the Zimbabwe DHS 2005–06 (left column), 2010–11 (middle column), and 2015 (right column). Model 1 (top row) and Model 2 (bottom row).

**Figure 4:**
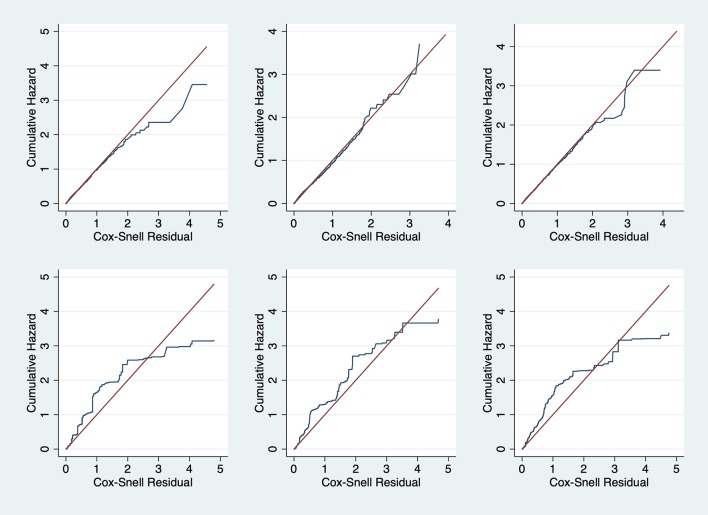
Cox-Snell residual against cumulative hazard goodness-of-fit tests for the Zimbabwe DHS 2005–06 (left column), 2010–11 (middle column) and 2015 (right column). Model 1 (top row) and Model 2 (bottom row).

**Figure 5:**
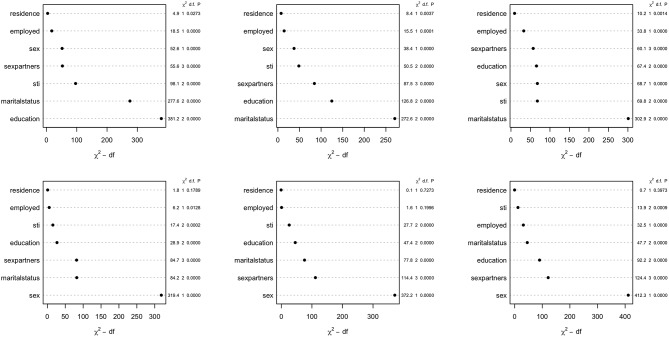
Dot chart showing relative importance of covariates for the Zimbabwe DHS 2005–06 (left column), 2010–11 (middle column), and 2015 (right column). Model 1 (top row) and Model 2 (bottom row).

The parametric Weibull distribution was used to investigate the factors associated with HIV infection. The Weibull parametric function was suitable to model the baseline hazard distribution, as shown in Figure 4. Model goodness of fit test indicated that Model 2 was the best model based on the Akaike Information Criterion (AIC) presented in Table 2. A graphical goodness of fit test was performed, where the semi-parametric model was compared to the parametric model. According to the results in Figure 3, Model 1 fits the data better than Model 1 and Model 2. Based on Figure 3, Model 1 overestimates the survival rates between 15 and 35 years and underestimates the survival rates between 35 and 49 years. However, Figure 4 with the Cox-Snell residuals shows that Model 1 is better than Model 2 as the estimated cumulative hazards are close to the reference line which is formed by the Cox-Snell residuals. Furthermore, dot charts depicting the importance of variables in the three models were plotted (Figure 5). Place of residence was the least important variable for all the three models, while marital status and sex where the most important variables for Model 1 and Model, respectively (Figure 5).

**Table 2:** Akaike information criterion and bayesian information criterion values.

**Year**	**Model**	**Obs**.	**ll(null)**	**ll(model)**	**df**	**AIC**	**BIC**
2005–06	1	9768	−2995.77	−2605.36	14	5238.7	5339.3
	2	9768	−2177.37	−1912.55	14	3853.09	3953.71
2010–11	1	10734	−2767.33	−2516.62	14	5061.23	5163.17
	2	10734	−2080.36	−1777.98	14	3583.95	3685.89
2015	1	12822	−2887.20	−2580.70	14	5189.41	5293.83
	2	12822	−2141.63	−1759.41	14	3546.83	3651.25

The risk of HIV infection was lower in males than females, as shown in Table 3 for all the models. The risk for HIV infection had a slight decrease from 2005–06 to 2010–11 to 2015 (HR = 0.26, 95% CI: 0.23, 0.31), (HR = 0.25, 95% CI:0.21,0.29), (HR = 0.22, 95% CI:0.19,0.26), respectively with reference to Model 2. Individuals who were married or cohabiting had a lower risk of HIV infection as compared to those who were single. These results were consistent for all three models. However, the risk of HIV infection for all the survey years was lower in individuals who were separated/divorced/widowed in reference to Model 1, but Model 2 results indicated that the risk was higher for that particular group of individuals as compared to those who were single (Table 3). According to the results in Model 1, the risk of HIV infection was almost the same for those who had one or more than two sexual partners, as compared to those who did not have any sexual partners in the past 12 months prior to the survey. However, Model 2, the risk was more than four times for those who had more than two sexual partners, as compared to those who did not have any sexual partners in the past 12 months prior to the survey. Of interest, the risk of HIV infection for those who had more than two sexual partners increased over time for Model 2, with Model 1 having a decreasing trend (Table 3).

**Table 3:** Estimated effects of covariates at baseline on the risk of HIV infection based on different survival models, Zimbabwe Demographic Health Survey (ZDHS) 2005/06, 2010/11, and 2015.

**Model**	**MODEL 1**			**MODEL 2**		
**Year**	**2005/06**	**2010/11**	**2015**	**2005/06**	**2010/11**	**2015**
**Variable**	**HR (CI)**	**HR (CI)**	**HR (CI)**	**HR (CI)**	**HR (CI)**	**HR (CI)**
**Sex**						
Female	1	1	1	1	1	1
Male	0.68 (0.62–0.76)	0.73 (0.66–0.80)	0.66 (0.60–0.73)	0.26 (0.23–0.31)	0.25 (0.21–0.29)	0.22 (0.19–0.26)
**Marital status**						
Single	1	1	1	1	1	1
Married/cohabiting	0.30 (0.25–0.35)	0.32 (0.27–0.38)	0.29 (0.25–0.34)	0.40 (0.33–0.50)	0.45 (0.37–0.55)	0.59 (0.48–0.71)
Separated/divorced/widowed	0.64 (0.54–0.76)	0.70 (0.59–0.84)	0.60 (0.50–0.71)	0.69 (0.55–0.87)	0.85 (0.67–1.08)	1.08 (0.84–1.38)
**Place of residence**						
Urban	1	1	1	1	1	1
Rural	1.11 (1.01–1.22)	0.87 (0.79–0.95)	0.86 (0.79–0.94)	1.10 (0.96–1.27)	1.03 (0.89–1.19)	1.06 (0.92–1.22)
**Education level**						
No education/primary	1	1	1	1	1	1
Secondary	2.53 (2.29–2.79)	1.62 (1.47–1.79)	1.05 (0.95–1.15)	1.12 (0.97–1.29)	0.91 (0.79–1.07)	0.63 (0.53–0.74)
Higher	1.32 (1.01–1.72)	0.75 (0.58–0.97)	0.51 (0.43–0.62)	0.43 (0.30–0.62)	0.30 (0.21–0.43)	0.25 (0.19–0.33)
**STI in the last 12 months**						
No	1	1	1	1	1	1
Yes	2.19 (1.88–2.56)	1.87 (1.57–2.22)	2.15 (1.79–2.58)	1.73 (1.33–2.25)	2.09 (1.58–2.76)	1.80 (1.31–2.46)
**No. of sexual partners**						
0	1	1	1	1	1	1
1	1.62 (1.42–1.86)	1.84 (1.61–2.12)	1.64 (1.43–1.89)	2.12 (1.71–2.64)	2.32 (1.86–2.89)	2.26 (1.77–2.88)
2+	1.95 (1.54–2.47)	2.19 (1.70–2.67)	2.06 (1.68–2.54)	4.91 (3.39–7.10)	5.58 (3.95–7.89)	6.88 (4.84–9.76)
**Currently employed**						
No	1	1	1	1	1	1
Yes	0.82 (0.75–0.90)	0.83 (0.76–0.91)	0.77 (0.70–0.84)	0.84 (0.74–0.96)	0.92 (0.80–0.99)	0.66 (0.57–0.76)

*HR, hazard ratio; CI, confidence interval*.

*Model 1 was the weibull parametric survival model with survey age imputation as the time and Model 2 was a weibull interval censoring model*.

## Discussion

Frequently, researchers are interested in using standard survival models in determining the failure times, however interval censoring has become increasingly common. The purpose of this study was to identify risk factors for HIV infection using three different models and determine the best fitting model. To identify the best fitting model, we utilized the Akaike Information Criterion (AIC), where the model with the least AIC value was the best fitting model. We also used graphical methods to ascertain the best fitting model, however graphical goodness-of-fit test for interval-censored data is rare with the available methods are still lacking in implementation (15–18). We managed to check the goodness-of-fit for all the models by overlaying the semi-parametric model with the fitted parametric model of the survival function and also plotting the Cox-Snell residuals against the cumulative hazard.

The model with interval censored data resulted in better estimates of the risk of HIV infection as compared to the standard survival model i.e., Models 1 based on the AIC value and Figure 3. The superiority of Model 2 was due to the ability to precisely mimic some of the results in literature from previous studies which also used population-based HIV surveys or specific cohorts in Zimbabwe. For example, a study in Zimbabwe conducted between 1999 and 2001 reported that they observed an increasing trend of HIV incidence among the educated individuals, which was rather unexpected; however, this might have been due to a higher socio-economic status, a factor reported to be associated with HIV infection in the region of Africa (19, 20). However, a systematic review which explored time trends in the association between educational attainment and risk of HIV infection in sub-Saharan Africa, reported a shift in the HIV epidemic from educated to the uneducated (21). According to a study in which they used the 2005/06 Zimbabwe Demographic Health Survey (ZDHS) to determine the relationship between HIV status and the demographic and socio-economic characteristics among adults in Zimbabwe by construction the risk profile of the average adult, they concluded that there was a significant negative association between HIV infection and education (22). They further clarified that an extra year of schooling to an average of 8 years (i.e., secondary education and above) was associated with a 0.5 percent point decrease in the probability of HIV infection for Zimbabwe (22). Another study which used the 2010/11 ZDHS also reported a lower risk of HIV infection of individuals with secondary level education and above (23). Based on these findings from literature, and the trend observed, the model with interval-censoring was consistent with the reported results, however, the other two models reported a higher risk of HIV infection for individuals with secondary level education, which was contrary to the findings from literature. according to an in-depth analysis of the 2005/06 ZDHS on the risk factors associated with HIV infection, it was reported that individuals who never had any sexually transmitted infection 12 months prior the survey were significantly associated with a 0.437 times lower risk of HIV infection compared with their counterparts who had a sexually transmitted infection during the same period (24). This result was close to the results obtained for the hazard of HIV infection in the model with interval-censoring. Another example was a study of the analysis of 2005/06 ZDHS, which reported that the likelihood of being HIV infected increased with the number of sexual partners and decreased with the level of faithfulness to a spousal partner. In the same study, it was reported that the odds of being HIV infected were 3 to 4 times greater among those who had two more sexual partner (25). These results were again consistent with results from the model with interval-censoring rather than the other two models for the 2005/06 ZDHS. On a general note, a 2013 study noted that having a large number of life partners increased HIV infection in a cohort from Manicaland (26).

Comparisons of the three models used in this study reveal a consistent match on the factors associated with HIV infection estimated from ZDHS data and the results obtained from previous studies using population-based HIV surveys or specific cohorts in Zimbabwe. For example, in all the three models, and based on all the three surveys, i.e., ZDHS 2005/06, 2010/11 and 2015, females were more at risk of HIV infection than men. Similarly, studies which determined the factors associated with HIV infection using the 2005/06 and 2010/11 ZDHS reported the same findings (22–24). However, though the surveys were the same, they did not use the same analytical methods to reach the same conclusions. Results suggest that marriage was associated with a lower risk of infection based on all the models. This is further supported by a study which analyzed the 2005/06 and 2010/11 ZDHS data (23). The study reported that marital union was positively associated with the decline of HIV infection for both men and women (23). Another study to determine the baseline predictions of HIV-1 acquisition among women reported that being unmarried was the strongest risk factor for HIV-1 acquisition (27). Results from these studies are again consistent with what all the models in our studies. Even though the models reported results similar to what had already been reported in literature, the precision of the model with interval-censoring in explaining some of the covariates is what stood out the most. However, the Cox-Snell residuals clearly showed that Model 1 was the best fitting model. The difference between the Cox-Snell residuals graphical method and the model estimates maybe due to the lack of adequate methods to test the goodness-of -fit of interval-censored data as cited by other authors (15–18).

The main strength of this study dependent on the quality of the data obtained from the surveys. These data were derived from population-based surveys, which in reality provides more reliable and robust data. Another strength of this study was due to the fact that we did not restrict our analysis to one method, however, we had the opportunity to determine the best model to fit the hazard of infection by comparing two different scenarios. For instance, if the median survival time for HIV infection was 5 years given the type of data we had, and the intervals were about 3 to 6 months wide, then we would have no reason to complicate the analysis by considering interval censoring. On the other hand, if the intervals were about 1 year or longer, then accounting for uncertainty in the analysis was necessary, which we did when we implemented the interval-censoring approach. Another reason for concluding that interval-censoring gave better estimates was due to its consistency with the published results from literature. Even though we consider the interval-censoring model as the superior model with regards to our specific data, the method had its own set of limitations. These limitations included the wide range of intervals used, which could have underestimated or overestimated the effect of other factors on the risk of HIV infection. Inclusion of competing risks factors in the model would have greatly improved the modeling approach. Further studies can be done on imputation models, which imputes an estimated time of HIV infection based on the data.

## Author Contributions

RB and EM conceived of the presented idea and verified the analytical methods and models. RB developed the theory and performed the data analysis. EM supervised the findings of this work. All authors discussed the results and contributed to the final manuscript.

### Conflict of Interest Statement

The authors declare that the research was conducted in the absence of any commercial or financial relationships that could be construed as a potential conflict of interest.
