# Supplementary material for: Factors Associated With HIV Infection in Zimbabwe Over a Decade From 2005 to 2015: An Interval-Censoring Survival Analysis Approach
Source: Front Public Health. 2019 Sep 18;7:262. doi: 10.3389/fpubh.2019.00262 (PMC6759818; doi:10.3389/fpubh.2019.00262)
Supplement: Supplementary file 1 [file Data_Sheet_1.docx]

### SUPPLIMENTARY DATA

### Model Specification

**Model 1**

Weibull regression model can be expressed as a proportional hazards model:

$$\lambda\left( t | \mathbb{X} \right)=\lambda_{0}\left( t \right)\exp\left( X_{1}\beta_{1}+\ldots+X_{k}\beta_{k} \right) (1)$$

where

$$\lambda_{0}(t)=exp(\beta_{0}){pt}^{p-1}$$

or

$\lambda\left( t | \mathbb{X} \right)={pt}^{p-1}$exp$\left( \mathbb{X}\beta\right) (2)$

where $\lambda\left( t | \mathbb{X} \right)$ is the hazard of getting HIV infection at age $t$ for a subject with covariate vector $\mathbb{X,}$ $\lambda_{0}(t)$ is the unspecified baseline hazard and $\beta$ is the vector of regression coefficient that measured the effect of $\mathbb{X}$ on the hazard of getting HIV infection, p is the Weibull shape parameter and $\pi=$ exp$(\mathbb{X}\beta)$ is the Weibull scale parameter.

**Censored Likelihood Function**

Suppose we have $n$ individuals all with observed events $t_{i}$ (no censoring), then the likelihood function is the product of the PDF:

$$L=\prod_{i=1}^{n} f(t_{i})$$

Given that they are individuals that are still HIV negative at the time the survey is taken, $t^{*}$, then the likelihood function is:

$$L=\prod_{t_{i}\leq t^{*}}^{n} f\left( t_{i} \right)\prod_{t_{i}>t^{*}}^{n} S\left( t_{i} \right), (3)$$

Therefore, the uncensored individuals contribute information to the likelihood function through the event times, and the censored individuals contribute information through the survival function at the end-point. This can be re-expressed as:

$$\sigma_{i}\left\{ \begin{matrix} 1 & if t_{i}\leq t^{*} \\ 0 & if t_{i}>t^{*} \end{matrix} \right.$$

With the likelihood function as:

$$\prod_{i=1}^{n} \left[ f\left( t_{i} \right) \right]^{\sigma_{i}} \left[ S\left( t_{i} \right) \right]^{1-\sigma_{i}} (4)$$

which shows the bias imposed by omitting right censored individuals.

### Model 2

We considered $t_{1}, \ldots,t_{n}$be the lifetimes for individuals from the 2005/06, 2010/11 and 2015 ZDHS sample of size $n$ where the probability density function (pdf) (for $t_{i}=t)$is represented by $f\left( t, \alpha, \beta\right),$the cumulative distribution function (cdf) is $F(t, \alpha, \beta)$ and the survival function is $S (t, \alpha, \beta)$ given the two-parameter Weibull distribution we have, respectively,

$f(t, \alpha,\beta$)=$\frac{\beta}{\alpha}\left( \frac{t}{\alpha} \right)^{\beta-1}exp\left( -\left( \frac{t}{\alpha} \right)^{\beta} \right), (5)$

$$F\left( t, \alpha,\beta\right)=1-exp\left( -\left( \frac{t}{\alpha} \right)^{\beta} \right), (6)$$

$$S\left( t, \alpha,\beta\right)=exp\left( -\left( \frac{t}{\alpha} \right)^{\beta} \right), (7)$$

Where $\alpha$ represents the scale parameter and $\beta$ the shape parameter.

Let $\left[ L_{i},R_{i} \right]$ denote the interval-censored data and let $T$ represent the unknown time of HIV infection, that is, $L_{i}\leq T_{i}\leq R_{i},$ where $L_{i}$is the age at first sexual intercourse and $R_{i}$ the age at survey date. If censoring occurs non-informatively and if the law governing $L$ and $R$ does not involve any of the parameters of interest, we can base our inferences on the likelihood function $L\left( L_{i},R_{i} \right|\alpha,\beta)$ as stated by Oller et al. ^10^, which is given by

$$L\left( L_{i},R_{i} \right| \alpha,\beta)=\prod_{i=1}^{n} \left[ F\left( R_{i},\alpha,\beta\right)-F\left( L_{i}, \alpha,\beta\right) \right]$$

$=\prod_{i=1}^{n} \left[ S\left( L_{i} \right)-S\left( R_{i} \right) \right] (8)$

$$=\prod_{i=1}^{n} prob\left\{ L_{i}\leq T_{i}\leq R_{i} \right\}.$$

Using (7), we have

$L\left( L_{i},R_{i} \right| \alpha,\beta)=\prod_{i=1}^{n} \left\{ exp\left( -\left( \frac{L_{i}}{\alpha} \right)^{\beta} \right)-exp\left( -\left( \frac{R_{i}}{\alpha} \right)^{\beta} \right) \right\}. (9)$

And taking the natural log of (9), we have

$\mathcal{l=}\sum_{i=1}^{n} ln\left\{ exp\left( -\left( \frac{L_{i}}{\alpha} \right)^{\beta} \right)exp\left( -\left( \frac{R_{i}}{\alpha} \right)^{\beta} \right) \right\}. (10)$

For us to find the values of $\alpha$ and $\beta$ that maximise (10), we differentiate the equation to obtain partial derivatives with respect to $\alpha$ and $\beta$ and set the resulting equations to zero.

Therefore,

$$\frac{\partial\mathcal{l}}{\partial\beta}=\sum_{i=1}^{n} - \frac{\left( \left( \frac{L_{i}}{\alpha} \right)^{\beta}In\left( \frac{L_{i}}{\alpha} \right)exp\left( -\left( \frac{L_{i}}{\alpha} \right)^{\beta} \right) \right)-\left( \left( \frac{R_{i}}{\alpha} \right)^{\beta}In\left( \frac{R_{i}}{\alpha} \right)exp\left( -\left( \frac{R_{i}}{\alpha} \right)^{\beta} \right) \right)}{exp\left( -\left( \frac{L_{i}}{\alpha} \right)^{\beta} \right)-exp\left( -\left( \frac{R_{i}}{\alpha} \right)^{\beta} \right)}=0$$

(11)

$$\frac{\partial\mathcal{l}}{\partial\alpha}=\sum_{i=1}^{n} -\frac{\left( \frac{L_{i}}{\alpha} \right)^{\beta}\left( \frac{\beta}{\alpha} \right)exp\left( -\left( \frac{L_{i}}{\alpha} \right)^{\beta} \right)-\left( \frac{R_{i}}{\alpha} \right)^{\beta}\left( \frac{\beta}{\alpha} \right)exp\left( -\left( \frac{R_{i}}{\alpha} \right)^{\beta} \right)}{exp\left( -\left( \frac{L_{i}}{\alpha} \right)^{\beta} \right)-exp\left( -\left( \frac{R_{i}}{\alpha} \right)^{\beta} \right)}=0 (12)$$

The maximum likelihood estimates of $\alpha$ and $\beta$ can then be obtained by using the Newton Raphson method.
